# Protective continuous ventilation strategy during cardiopulmonary bypass in children undergoing surgery for congenital heart disease: a prospective study

**DOI:** 10.1093/icvts/ivac084

**Published:** 2022-03-25

**Authors:** Massimo A Padalino, Luca Vedovelli, Manuela Simonato, Andrea Bandini, Greta Paganini, Laura Mezzalira, Nicola Faganello, Cristiana Carollo, Dario Gregori, Vladimiro Vida, Paola Cogo

**Affiliations:** 1 Pediatric and Congenital Cardiac Surgery Unit, Department of Cardiac, Thoracic, and Vascular Sciences, and Public health, University of Padova, Padova, Italy; 2 Unit of Biostatistics, Epidemiology, and Public Health, Department of Cardiac, Thoracic, and Vascular Sciences, and Public health, University of Padova, Padova, Italy; 3 PCare Laboratory, Fondazione Istituto di Ricerca Pediatrica Città della Speranza, Padova, Italy; 4 Anesthesia and Resuscitation Institute, Department of Medicine DIMED, University of Padova, Padova, Italy; 5 Division of Pediatrics, Department of Medicine, University Hospital Santa Maria della Misericordia, University of Udine, Udine, Italy

**Keywords:** Cardiopulmonary bypass, Ventilation, Systemic inflammatory response syndrome, Lung injury, Congenital heart disease, Paediatric

## Abstract

**OBJECTIVES:**

The aim of this study was to evaluate if a ‘protective’ (low-tidal/low-frequency) ventilation strategy can shorten the postoperative ventilation time and minimize acute lung injury in children with congenital heart disease (CHD) undergoing repair with cardiopulmonary bypass (CPB).

**METHODS:**

This is a single-centre prospective, interventional study, including children with CHD under the age of 5 years, undergoing open-heart surgery with a CPB >60 min, in hypothermia, haemodynamically stable, and without evident genetic abnormalities. Assist-control ventilation (tidal volume of 4 ml/kg, 10 breaths/min, positive end-expiratory pressure 5 cmH_2_O and FiO_2_ 0.21) was applied in a cohort of patients during CPB. We compared clinical outcomes and in fully ventilated versus non-ventilated (control) patients. Propensity score was used to weigh ventilated and control groups to correct for the effect of other confounding clinical variables. Clinical and ventilation parameters and lung inflammatory biomarkers in tracheal aspirates were measured. The primary outcome was the postoperative intubation time of more or less than 48 h.

**RESULTS:**

We included 140 children (53 ventilated, 87 non-ventilated) with different CHD. There were no deaths or adverse events in ventilated patients. Using a weighted generalized linear model, we found no sufficient evidence for an effect of intraoperative ventilation on postoperative intubation time [estimate 0.13 (95% confidence interval, –0.08; 0.35), *P* = 0.22].

**CONCLUSIONS:**

Continuous low-tidal/low-frequency mechanical ventilation during CPB is safe and harmless. However, no significant advantages were found when compared to non-ventilated patients in terms of postoperative ventilation time.

**Figure:**
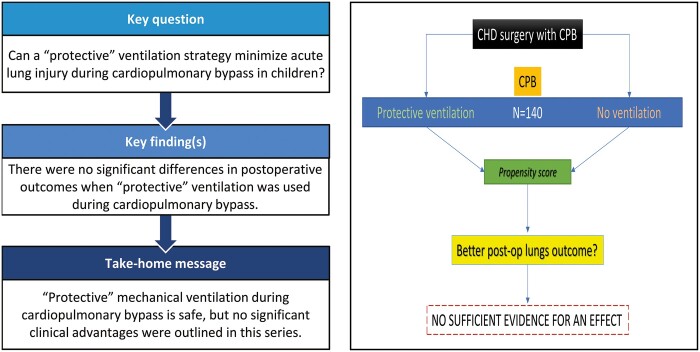

## INTRODUCTION

Pulmonary dysfunction after cardiac surgery with cardiopulmonary bypass (CPB) can affect the overall outcome of children with congenital heart diseases (CHD). Prolonged lung deflation can accentuate the systemic inflammatory response syndrome (SIRS) caused by the blood–CPB circuit surface contact, and non-pulsatile flow, and can influence negatively the early postoperative course in the cardiac intensive care unit (CICU) [1]. Hypoxia, atelectasis [2], increase of intrapulmonary shunts [3], increase of capillary permeability [4], gas exchange alteration, and increased infection susceptibility are described as pulmonary complications induced by CPB that could result in an overall worse outcome.

Several techniques have been studied to minimize or, possibly, eliminate post-CPB injuries. Most of them are focused on reducing inflammation by administering drugs, such as steroids, statins or heparin [5, 6]. Non-pharmacological strategies are focused on CPB miniaturization, and utilization of biocompatible materials and blood filters [7, 8]. Most of these strategies have shown initial promising results, such as lower cytokine production and reactive oxygen species formation, but they have mostly failed to prove a better clinical outcome [9, 10]. Systemic inflammatory lesions induced by CPB are even more evident in the paediatric population, where clinical studies are lacking.

Low-tidal, low-frequency mechanical ventilation during CPB is an inexpensive and easy-to-apply strategy that can be used to avoid prolonged lung deflation in cardiac surgical patients. The rationale of this intervention is the hypothesized beneficial effects of a para-physiologic (a.k.a. ‘protective’) ventilation, which can warrant normally inflated lung parenchyma during CPB. Currently, there are not enough data to support or negate this hypothesis.

This study aimed to evaluate whether a continuous low-tidal/low-frequency mechanical ventilation strategy during CPB in children with CHD undergoing surgical repair could effectively minimize the acute lung injury (protect) and possibly improve postoperative outcomes. This hypothetical improvement was objectively evaluated by assessing the length of postoperative ventilation time and cardiac intensive care unit (ICU) stay and dosing the lung inflammation markers in the preoperative and early postoperative tracheal aspirate (TA).

## MATERIALS AND METHODS

### Patients

This is a prospective, single-centre study in children with complex CHD undergoing a cardiac surgical procedure with CPB from 2017 to 2019. Included were children undergoing a surgical procedure with a CPB time >60 min, and an aortic cross-clamp time >20 min (when performed), on hypothermia (body core temperature <35°C). Exclusion criteria were: age >5 years, associated chromosomal abnormalities (e.g. trisomy 21), preoperative unstable haemodynamic condition, factor V <20% or creatinine clearance <30% before surgery and need of CPB or ECMO for an early unexpected reoperation (within 30 days).

### Ethics statement

The study was approved by the local Ethics Committee on Clinical Investigation (prot. number 3142/AO/14) and registered in ClinicalTrials.gov (NCT03255356). Informed consent was obtained from all the study participants’ parents or tutors.

### Surgery and ventilation

Anaesthesia and CPB management were described in detail previously [11]. Briefly, after anaesthesia induction and heparin administration, patients were cannulated, and CPB was initiated with a haematic prime to keep hematocrit between 25% and 30%. Children were ventilated during CPB on assist-control pressure mode, at 10 breaths/min, tidal volume 4 ml/kg, positive end-expiratory pressure (PEEP) 5 cmH_2_O, and FiO_2_ 0.21. The decision to ventilate or not ventilate the patient was planned to be randomly assigned. However, it was expected that the ventilation could be modified if inflation and deflation of the lungs interfered with the operative field or patient's safe CPB period. Deep hypothermic cardiac arrest or selective regional cerebral perfusion were applied according to the type of procedure performed; CPB flows were set according to body surface area, cardiac index, and temperature nadir. Blood gas analysis and metabolic parameters were measured at least every 20 min. At the end of surgery, all patients underwent rewarming to 36°C, followed by modified ultrafiltration. Then, they were transferred to the CICU and followed during the postoperative course until extubation and CICU discharge, or for the first 24 postoperative hours.

TAs were collected immediately after anaesthesia induction, at intubation, and at the end of cardiac surgery, after chest closure. The procedure for TAs collection was standardized as described previously [12]. One hundred microlitres of fluid were stored untouched at −80°C for myeloperoxidase (MPO) activity analysis, and the rest was centrifuged at 400 × *g* for 10 min to sediment cells and cell debris. Aliquots of 400 microliters of supernatants were stored at −80°C. At the same time as TAs collection, 100 µl of fresh whole blood were drawn into EDTA-containing tubes, centrifuged at 1400 × *g* for 10 min, to store the plasma at −80°C until analysis. Samples with evident blood stains or haemolysis were discarded.

Cardiac surgical procedures were stratified according to STAT (The Society of Thoracic Surgeons-European Association for Cardio-Thoracic Surgery) categories [13]. Postoperative adverse events (AE) were anticipated as pneumothorax, air leak, pleural effusion, cardiac arrhythmia and cardiac arrest.

### Sample analysis

Albumin concentration was measured with the bromocresol green method [14], and MPO was measured in whole TA as previously reported [15]. TA dilution was calculated by analysing plasma and TA urea levels using a commercial kit (QuantiChrom urea assay kit; Bioassay System, Hayward, CA, USA). The ratio between plasma urea and TA urea reflected TA dilution [16].

### Statistical methods

#### Sample size estimation

The sample size computation has been performed via Monte Carlo simulation procedure consisting of 1000 runs. For each run, the data have been simulated by assuming the effect of 5 standardized Normal covariates on the probability of being mechanically ventilated to mimic the lack of randomization effect on the group assignment. The group assignment has been simulated via the binomial logit function. The treatment effect has been assumed equal to an odds ratio of 2.5 corresponding to a high Cohen *D* effect size of 0.8. The outcome variable has been generated with a binomial logit function. For each simulated data the *P*-value for the intervention effect has been estimated with an inverse probability of treatment weighting (IPTW) logistic regression model. A 0.8 Cohen *D* effect size led to achieving a significant intervention effect on the 70% of simulations with a sample size of 125 patients.

#### Statistical analysis

We designed a prospective observational analysis using propensity score. A randomized trial could not be conducted due to the complexity of recruitment and peculiarity of the single surgeries. We aimed to have a ratio of 2:1 between non-ventilated and ventilated patients for each STAT score to have comprehensive view of all CHDs. Group assignment was conducted using a pseudo-randomization from the laboratory unit (blinded towards the specific patient conditions, unless for its STAT score) to obtain roughly the same number of ventilated/non-ventilated patients in each STAT category. For example, for the first 3 prospectively recruited patients in the STAT 1 category, the first 2 were not ventilated, and the last one was ventilated.

Data are expressed as median and interquartile ranges. To verify the effect of mechanical ventilation, we applied a propensity score approach to compare treated and control patients minimizing the confounding effects of covariates or outcomes on the clinical question. Specifically, we used the IPTW that uses the probability of being ventilated (treated) as the weight assigned to every single patient [17]. Variables used for propensity score and subsequent IPTW were selected based on their clinical significance (explained in the parentheses after the variable): weight (for normal development and also used as a proxy of age), sex, presurgery TA MPO activity (to account for lung inflammation), presurgery PaO_2_/FiO_2_ ratio (for gas exchange), presurgery pCO_2_, STAT category (to account for CHD complexity), CPB duration and CPB temperature nadir. We considered postoperative intubation of more or less than 48 h as the primary outcome. The difference in intubation time between the ventilated and non-ventilated groups was assessed with a weighted generalized linear model with design-based standard errors, with a single covariate (ventilated or non-ventilated). The packages {MatchIt}, {WeighIt} and {survey} of R software (v. 4.1.1) were used for the analysis. The full code and dataset are available at https://osf.io/yf95a/.

## RESULTS

**Figure 1:**
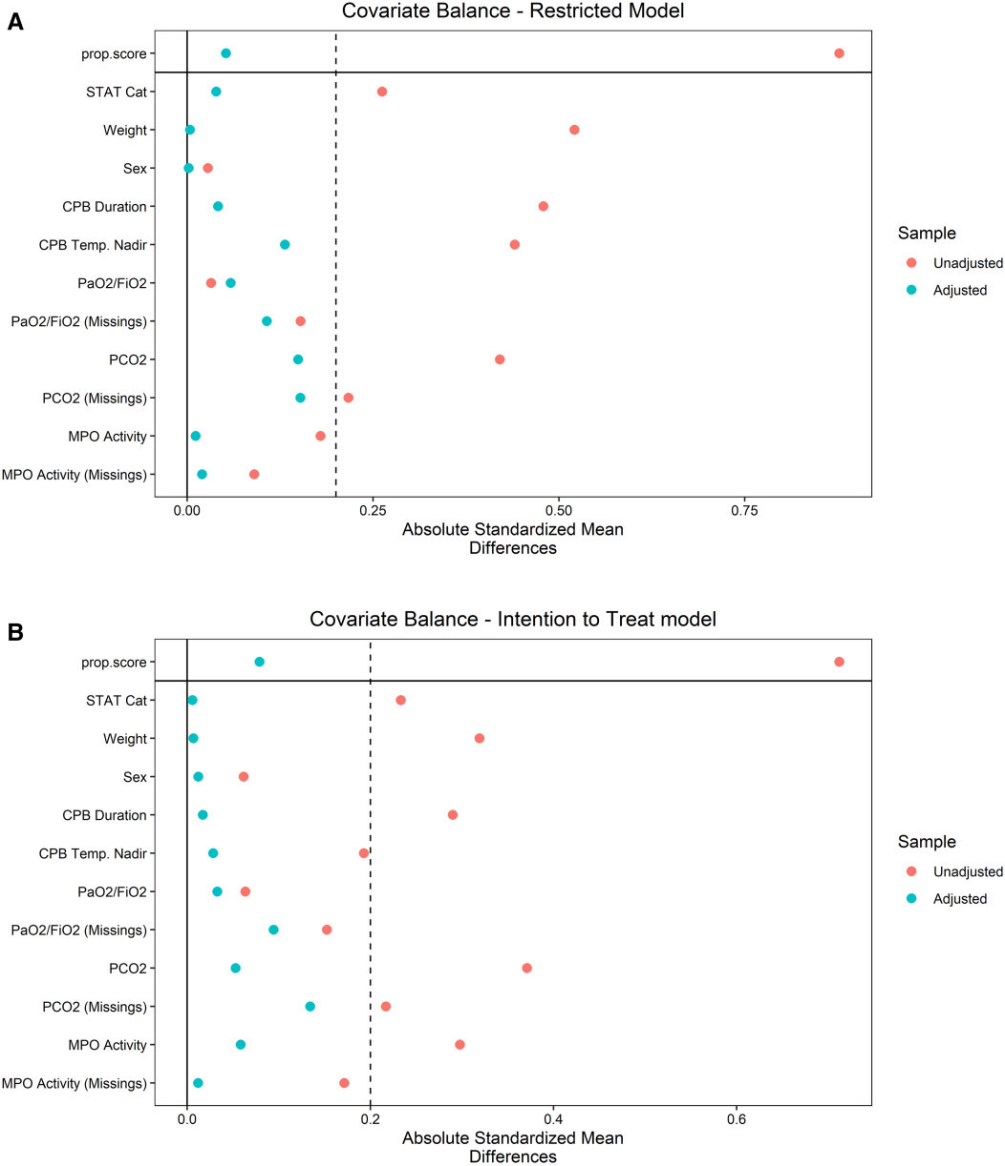
Absolute mean differences for propensity score variables. Variables are shown before (‘unadjusted’) and after (‘adjusted’) propensity score weighting. Missing values are also weighted in the variables that have some. Panel (**A**) refers to the restricted model (only patients that completed the ventilation on cardiopulmonary bypass procedure were included) and panel (**B**) refers to the intention-to-treat model (all patients were included. Patients partially ventilated during cardiopulmonary bypass were included in the ventilated group). The absolute standardized mean difference is calculated as the absolute value in the difference in means of a variable across the treatment groups, divided by the standard deviation in the treated group. It is a measure of the effect size of the differences.

We prospectively enrolled 140 children (ventilated 53, controls 87). Fifteen patients were switched from protective ventilation to conventional no-ventilation upon request of the surgeons to optimize the surgical view by decreasing pulmonary venous return. Therefore, we conducted the same analysis twice: (i) a ‘restricted model’ group, including only ventilated patients that completed the CPB ventilation procedure (*n* = 38), versus non-ventilated (*n* = 87) and (ii) ‘intention-to-treat (ITT) model’ group, including all ventilated patients (even those patients who were partially ventilated, a.k.a. ITT patients, *n* = 53), versus non-ventilated (*n* = 87). Patients' preoperative characteristics are summarized in Table 1. Most patients were affected by septal defects (58 patients), followed by tetralogy of Fallot-type defects (36) and transposition of the great arteries (26). After propensity score, the absolute standardized mean difference was ≤0.2 for all the matching variables (Figure 1).

**Table 1:** Preoperative characteristics

Characteristic	Overall	Non-ventilated	Fully ventilated	Partially ventilated
	*N* = 140^a^	*N* = 87^a^	*N* = 38^a^	*N* = 15^a^
Age (months)	4.1 (2.1, 8.3)	3.7 (1.7, 7.0)	6.2 (3.2, 36.8)	3.9 (0.3, 5.2)
Sex (male)	66 (47)	40 (46)	18 (47)	8 (53)
Weight (kg)	5.3 (3.9, 7.1)	5.0 (3.8, 6.8)	6.6 (4.7, 12.0)	4.8 (3.5, 6.1)
Neonates	29 (21)	19 (22)	4 (11)	6 (40)
CHD type				
TGA	26 (19)	15 (17)	5 (13)	6 (40)
TOF/DORV TOF like	36 (26)	29 (33)	3 (7.9)	4 (27)
VSD (large or multiple)	58 (41)	32 (37)	21 (55)	5 (33)
HLHS	4 (2.9)	3 (3.4)	1 (2.6)	0 (0)
Single ventricle	11 (7.9)	5 (5.7)	6 (16)	0 (0)
TAPVR	5 (3.6)	3 (3.4)	2 (5.3)	0 (0)
Pre-op. pCO_2_ (mmHg)	33 (28, 39)	33 (27, 38)	34 (30, 40)	32 (30, 42)
Pre-op. pO_2_ (mmHg)	92 (56, 182)	91 (56, 168)	108 (58, 189)	58 (52, 136)
Pre-op. oxygen saturation (%)	96 (87, 99)	97 (87, 99)	98 (90, 100)	94 (87, 100)
Pre-op. PaO_2_/FiO_2_	438 (267, 869)	435 (265, 802)	515 (276, 901)	278 (248, 647)
Pre-op. TA MPO (mU/ml)	284 (78, 854)	359 (64, 1048)	273 (89, 792)	131 (72, 154)
Pre-op. TA albumin (mg/ml)	10 (6, 16)	9 (5, 15)	13 (8, 16)	10 (7, 17)

Unknown: missing data. Continuous variables are expressed as median (interquartile range). A full table with missing data description is available as Supplementary Material, Table S1.

a Median (IQR) and *n* (%).

CHD: congenital heart disease; DORV: double-outlet right ventricle; IQR: interquartile range; MPO: myeloperoxydase activity; TA: tracheal aspirate; TAPVR: total anomalous pulmonary venous return; TGA: transposition of the great arteries; TOF: tetralogy of Fallot; VSD: ventricular septal defect; HLHS: hypoplastic left heart syndrome; EDTA: Ethylenediaminetetraacetic acid.

All intraoperative and postoperative data are reported in Table 2. During the postoperative observation, there were no AE that could be correlated to the intraoperative ventilation strategies. All patients survived until hospital discharge.

**Table 2:** Intraoperative and postoperative characteristics

Characteristic	Overall	Non-ventilated,	Fully ventilated	Partially ventilated
	*N* = 140^a^	*N* = 87^a^	*N* = 38^a^	*N* = 15^a^
STAT				
1	20 (14)	13 (15)	6 (16)	1 (6.7)
2	71 (51)	40 (46)	23 (61)	8 (53)
3	27 (19)	17 (20)	4 (11)	6 (40)
4	20 (14)	15 (17)	5 (13)	0 (0)
5	2 (1.4)	2 (2.3)	0 (0)	0 (0)
Surgery time (min)	210 (180, 245)	220 (191, 240)	198 (156, 245)	225 (210, 252)
CPB time (min)	114 (80, 138)	122 (90, 138)	86 (68, 115)	121 (108, 154)
Aortic cross-clamp (min)	57 (39, 78)	60 (45, 82)	44 (28, 67)	65 (55, 96)
Temperature nadir (°C)	32.0 (30.0, 33.1)	32.0 (30.0, 32.5)	33.0 (32.0, 34.0)	29.5 (27.3, 32.5)
Hypothermia (min)	70 (50, 96)	70 (60, 98)	56 (30, 80)	100 (70, 110)
Rewarming (min)	25 (20, 30)	30 (20, 30)	20 (15, 30)	26 (20, 30)
DHCA	3 (2.2)	2 (2.3)	1 (2.6)	0 (0)
CPB pH	7.40 (7.36, 7.44)	7.39 (7.37, 7.42)	7.40 (7.36, 7.44)	7.42 (7.37, 7.47)
CPB lactates (mmol/l)	1.88 (1.50, 2.50)	1.88 (1.50, 2.78)	1.90 (1.46, 2.28)	1.79 (1.49, 2.65)
CPB pCO_2_	34.3 (30.4, 37.1)	34.7 (31.0, 36.9)	34.3 (29.1, 37.9)	33.2 (28.9, 35.2)
CPB pO_2_	169 (144, 197)	168 (145, 194)	170 (130, 198)	169 (150, 197)
Post-surgery PaO_2_/FiO_2_	174 (90, 332)	168 (85, 296)	174 (106, 416)	241 (102, 356)
Post-surgery TA MPO (mU/ml)	844 (322, 2113)	1113 (440, 2671)	714 (266, 1890)	693 (240, 1089)
Post-surgery TA albumin (mg/ml)	10 (7, 14)	10 (6, 14)	10 (8, 15)	12 (10, 24)
Post-surgery mechanical ventilation (h)	36 (27, 73)	34 (28, 76)	30 (11, 54)	54 (32, 74)
Post-surgery mechanical ventilation >48 h	65 (46)	40 (46)	16 (42)	9 (60)
ICU stay (days)	2.66 (1.65, 4.66)	3.65 (1.65, 4.67)	2.65 (1.65, 3.65)	3.65 (2.66, 5.39)
Length of stay (days)	8.7 (6.6, 13.2)	8.6 (6.6, 12.7)	9.6 (6.6, 13.6)	7.6 (6.7, 11.7)

Unknown: missing data. Continuous variables are expressed as median (interquartile range). A full table with missing data description is available as Supplementary Material, Table S2.

a *n* (%) and median (IQR).

CPB: cardiopulmonary bypass; DHCA: deep hypothermic cardiac arrest; ICU: intensive care unit; IQR: interquartile range; MPO: myeloperoxydase activity; STAT: The Society of Thoracic Surgeons-European Association for Cardio-Thoracic Surgery score; TA: tracheal aspirates.

The generalized linear models showed no evidence for a difference in the postoperative ventilation time in ICU between groups, calculated both as more or less than 48 h intubation:

- Restricted model estimate 0.13 (95% confidence interval, −0.08; 0.35), *P* = 0.22 and
- ITT model estimate 0.11 (95% confidence interval, −0.07 to 0.29; 0.10), *P* = 0.25.

Last, even if there was a positive trend, we could not demonstrate a significant difference in postoperative MPO or TA albumin between groups evaluated with the same model (data and code are publicly available at https://osf.io/yf95a/).

## DISCUSSION

In this study, we aimed to evaluate whether a continuous mechanical ventilation with low volumes/low respiratory rate could ‘protect’ the lungs from SIRS and improve clinical outcomes (postoperative ventilation time), in paediatric patients undergoing repair of complex CHD undergoing on CPB.

As described elsewhere [18], SIRS is considered one of the main causes of postoperative lung dysfunction, which is mainly triggered by lung deflation and loss of aerated alveoli when ventilation is off [19]. The cardiovascular and respiratory systems are closely interdependent in children with CHD, and blood flow may vary according to the pulmonary-to-systemic vascular resistance ratio. Depending on the CHD, lungs may be exposed to increased or decreased pulmonary blood flow (i.e. ventricular septal defect or tetralogy of Fallot, respectively) [20]. Also, TA's composition has distinctive patterns in different CHD [21]. In fact, preoperatively, surfactant-specific protein SP-B is increased in all CHD, while MPO (neutrophil) activity is decreased only in tetralogy of Fallot, and albumin and surfactant-specific protein-A are increased only in ASD and ventricular septal defect, compared to age-matched controls [21].

During CPB in surgery for CHD, SIRS can trigger the development of an acute lung injury, especially in neonates and children since their pulmonary vascularization is more reactive and prone to pulmonary hypertension [22, 23]. Moreover, CPB circuits require extensive blood dilution for the circuit-priming volume, which is partially corrected with haematic prime [24]. Thus, these 2 factors together can increase the risk of pulmonary oedema and hypertension in newborns and infants [25].

Mechanical ventilation during CPB in adults is proven to be safe, although advantages are still debateable [26]. Recently, a meta-analysis on 17 trials, including 1162 adult patients, showed that ventilation during CPB might improve post-CPB oxygenation and gas exchange in adult cardiac surgical patients, but there is no evidence of long-term beneficial effects [26]. In the paediatric field, very few studies have been reported. Sasson *et al.* [27] compared 5 types of mechanical ventilation during CPB in 50 children without significant evidence of clinical improvement in ventilated patients. However, major limitations of this report were the a too wide age range (0.3–15 years), and timing of outcome measurements (5 min after sternal closure), with no mid or long-term follow-up.

In our experience, we have adopted a low-tidal/low-frequency mechanical ventilation strategy as it is the safest and possibly more protective modality of intra-CPB ventilation. In particular, pressure control ventilation may warrant adequate control of peak pressure and avoidance of barotrauma. However, it requires more careful patient monitoring to avoid the risk of hypoventilation if airways resistance should increase. In our practice, we used a 4 ml/kg tidal volume associated with modest hyperventilation to keep pCO_2_ in the 35–40 mmHg range, which can suppress the spontaneous ventilation trigger and improve adaptation to mechanical ventilation. Our policy was to keep pO_2_ always inferior to 60% to avoid O_2_ toxicity, with the possible aid of nitric oxide in case of hypoxia. In the paediatric age, PEEP is suggested since it is more effective in preventing alveolar and small bronchi collapse, minimizing atelectasis. Also, it reduces the migration of interstitial fluid to the alveoli and prevents oedema. In our hands, this approach was a safe, inexpensive and feasible technique with no related AE. Of note, as a side effect, this ventilation mode can increase blood return from pulmonary veins and occasionally obscure the surgical field in newborns. A temporary suspension of the continuous ventilation may be necessary. In our experience, this happened in 15 patients who were excluded from the ‘restricted’ group, whose age and weight were minor (neonates in 40%), and whose surgical complexity was slightly higher than the included patients (STAT 2 and 3 in about 90%). Thus, this is not an issue in paediatric patients undergoing other than intracardiac (i.e. intraatrial or intraventricular) repair, such as cavopulmonary anastomosis or aortic arch reconstruction. It is of note that in most cases, the low-tidal low-frequency ventilation strategy did not cause any interference in bigger infants. However, alternative or larger venting cannulas may be used to minimize blood flooding.

In this study, we could not find any significant difference either in intubation time and ICU stay or in lung inflammatory biomarkers. We focused our study either on clinical or biochemical outcomes to prove that this strategy could minimize lung deflation, alveolar collapse and pulmonary inflammation. We found that the median time of mechanical ventilation and intensive care stay were, respectively, 4 h and 1 day less in the fully ventilated group. The respective interquartile ranges were persistently lower in the ventilated group. Although not statistically significant, we believe that these results could be clinically relevant, and a larger experience may confirm our impression. Compared to other studies [27], ours is a prospective analysis in which we collected a homogeneous cohort of patients <5 years of age with similar types of CHD. We introduced the use of the IPTW technique to minimize the selection bias due to the dropouts and verify the robustness of our data regarding the claimed protective role of CPB ventilation on the total postoperative ventilation time.

Interestingly, these findings were associated with a reduced MPO activity (although not significantly) in both groups (Table 2). On the contrary, TA albumin (an index of capillary permeability) did not show any difference. An increase in MPO during CPB has already been reported [28]. It is well known that ischaemia during CPB results in endothelial activation upon reperfusion [29]. During this phase, the mismatch between pulmonary oxygen demand and supply can result in oxidative stress, in which excessive reactive oxygen species accumulate. Thus, a tendency of a reduction of MPO may be evidence of a decreased inflammatory injury enhanced by a protective ventilation strategy. However, further experience with larger studies is needed to support or refute this hypothesis.

Last, we believe that it is important to underline that the routine anaesthesiological manouvres before weaning from CBP were performed by different physicians. Thus, various anaesthesiologists have been using as a re-expansion method the common manual compression of the self-inflating bag in the ventilator’s circuit, often unwillingly forcefully, until the lungs appear fully expanded. This is certainly quite traumatic and may have affected the protective effect of the continuous mechanical ventilation, whose aim was to avoid atelectasis, which itself causes inflammation and surfactant loss. A much gentler strategy such as gradual increase of inspiratory pressure and PEEP may minimize the trauma of re-expansion. The manual compression recruiting was considered by our anaesthesiology staff as a quicker and more adequate de-airing strategy, when compared to other methods (i.e. the incremental PEEP). However, thanks to this study, we have learned the lesson and we have modified it.

### Limitations

There are some important limitations to this study. Most relevant one is probably the manual recruiting manoeuvre that has been often used before weaning from CPB and may have influenced the outcomes. Second, even if corrected with an IPTW methodology, there is a conspicuous patient selection bias since a randomized enrolment could not be done as originally planned. Last, being a single-centre study, the ventilation modalities and the extubation criteria may not be universally accepted. We also noticed that our hypothesis of a high effect size of 0.8 was overestimated. This could have led to a lower real statistical power than the one expected during the study design. We also think that subgroups response could have been unbalanced (i.e. some STAT score groups responded worse than others) but the sample size did not permit a subgroup analysis with enough power.

However, this study is a prospective one, with a homogeneous population (age, type of CHD), and even if not completely randomized, it was conducted using the best data analysis practices to avoid systematic biases.

## CONCLUSION

Continuous low-tidal/low-frequency mechanical ventilation strategy during CPB is safe and feasible, with no harm to paediatric patients. We did not find sufficient evidence for a difference in intubation time and ICU stay, or inflammation biomarkers expression during the first 24 postoperative hours. However, further experience, with a larger sample of patients and possibly a multicentric study, and less harmful lung re-expansion techniques may confirm these preliminary results.

## SUPPLEMENTARY MATERIAL

Supplementary material is available at *ICVTS* online.

## Supplementary Material

ivac084_Supplementary_Data

## ABBREVIATIONS

AE: Adverse events
CHD: Congenital heart disease
CICU: Cardiac intensive care unit
CPB: Cardiopulmonary bypass
ICU: Intensive care unit
IPTW: Inverse probability of treatment weighting
ITT: Intention to treat
MPO: Myeloperoxidase
PEEP: Positive end-expiratory pressure
SIRS: Systemic inflammatory response syndrome
TA: Tracheal aspirate
