# Supplementary material for: Protective continuous ventilation strategy during cardiopulmonary bypass in children undergoing surgery for congenital heart disease: a prospective study
Source: Interact Cardiovasc Thorac Surg. 2022 Mar 25;35(2):ivac084. doi: 10.1093/icvts/ivac084 (PMC9297524; doi:10.1093/icvts/ivac084)
Supplement: ivac084_Supplementary_Data [file ivac084_supplementary_data.docx]

**Table S1:** Pre-operative characteristics

| Characteristic | Overall  N = 140^1^ | Non-Ventilated  N = 87^1^ | Fully Ventilated  N = 38^1^ | Partially Ventilated  N = 15^1^ | |
| --- | --- | --- | --- | --- | --- |
| Age (months) | 4.1 (2.1, 8.3) | 3.7 (1.7, 7.0) | 6.2 (3.2, 36.8) | 3.9 (0.3, 5.2) |  |
| Sex (male) | 66 (47%) | 40 (46%) | 18 (47%) | 8 (53%) |  |
| Weight (kg) | 5.3 (3.9, 7.1) | 5.0 (3.8, 6.8) | 6.6 (4.7, 12.0) | 4.8 (3.5, 6.1) |  |
| Neonates | 29 (21%) | 19 (22%) | 4 (11%) | 6 (40%) |  |
| CHD type |  |  |  |  |  |
| *TGA* | 26 (19%) | 15 (17%) | 5 (13%) | 6 (40%) |  |
| *TOF/DORV TOF like* | 36 (26%) | 29 (33%) | 3 (7.9%) | 4 (27%) |  |
| *VSD (large or multiple)* | 58 (41%) | 32 (37%) | 21 (55%) | 5 (33%) |  |
| *HLHS* | 4 (2.9%) | 3 (3.4%) | 1 (2.6%) | 0 (0%) |  |
| *Single Ventricle* | 11 (7.9%) | 5 (5.7%) | 6 (16%) | 0 (0%) |  |
| *TAPVR* | 5 (3.6%) | 3 (3.4%) | 2 (5.3%) | 0 (0%) |  |
| Pre-op. pCO2 (mmHg) | 33 (28, 39) | 33 (27, 38) | 34 (30, 40) | 32 (30, 42) |  |
| Unknown | 2 | 2 | 0 | 0 |  |
| Pre-op. pO2 (mmHg) | 92 (56, 182) | 91 (56, 168) | 108 (58, 189) | 58 (52, 136) |  |
| Unknown | 1 | 1 | 0 | 0 |  |
| Pre-op. Oxygen saturation (%) | 96 (87, 99) | 97 (87, 99) | 98 (90, 100) | 94 (87, 100) |  |
| Unknown | 2 | 2 | 0 | 0 |  |
| Pre-op. PaO2/FiO2 | 438 (267, 869) | 435 (265, 802) | 515 (276, 901) | 278 (248, 647) |  |
| Unknown | 1 | 1 | 0 | 0 |  |
| Pre-op. TA MPO (mU/ml) | 284 (78, 854) | 359 (64, 1,048) | 273 (89, 792) | 131 (72, 154) |  |
| Unknown | 21 | 11 | 6 | 4 |  |
| Pre-op. TA albumin (mg/ml) | 10 (6, 16) | 9 (5, 15) | 13 (8, 16) | 10 (7, 17) |  |
| Unknown | 28 | 18 | 4 | 6 |  |
| ^1^Median (IQR); n (%) | | | | |  |

Legend: CHD: Congenital heart disease; DORV: double-outlet right ventricle; MPO: Myeloperoxydase activity; TA: tracheal aspirate; TAPVR: total anomalous pulmonary venous return; TGA: transposition of the great arteries; TOF: tetralogy of Fallot; VSD: ventricular septal defect; Unknown: missing data. Continuous variables are expressed as median (interquartile range)

**Table S2:** Intraoperative and post-operative characteristics

| Characteristic | Overall  N = 140^1^ | Non-Ventilated,  N = 87^1^ | Fully Ventilated  N = 38^1^ | Partially Ventilated  N = 15^1^ |
| --- | --- | --- | --- | --- |
| STAT |  |  |  |  |
| *1* | 20 (14%) | 13 (15%) | 6 (16%) | 1 (6.7%) |
| *2* | 71 (51%) | 40 (46%) | 23 (61%) | 8 (53%) |
| *3* | 27 (19%) | 17 (20%) | 4 (11%) | 6 (40%) |
| *4* | 20 (14%) | 15 (17%) | 5 (13%) | 0 (0%) |
| *5* | 2 (1.4%) | 2 (2.3%) | 0 (0%) | 0 (0%) |
| Surgery time (min) | 210 (180, 245) | 220 (191, 240) | 198 (156, 245) | 225 (210, 252) |
| Unknown | 1 | 1 | 0 | 0 |
| CPB time (min) | 114 (80, 138) | 122 (90, 138) | 86 (68, 115) | 121 (108, 154) |
| Aortic cross-clamp (min) | 57 (39, 78) | 60 (45, 82) | 44 (28, 67) | 65 (55, 96) |
| Unknown | 2 | 0 | 2 | 0 |
| Temperature nadir (°C) | 32.0 (30.0, 33.1) | 32.0 (30.0, 32.5) | 33.0 (32.0, 34.0) | 29.5 (27.3, 32.5) |
| Hypothermia (min) | 70 (50, 96) | 70 (60, 98) | 56 (30, 80) | 100 (70, 110) |
| Unknown | 1 | 1 | 0 | 0 |
| Rewarming (min) | 25 (20, 30) | 30 (20, 30) | 20 (15, 30) | 26 (20, 30) |
| Unknown | 1 | 1 | 0 | 0 |
| DHCA | 3 (2.2%) | 2 (2.3%) | 1 (2.6%) | 0 (0%) |
| Unknown | 1 | 0 | 0 | 1 |
| CPB pH | 7.40 (7.36, 7.44) | 7.39 (7.37, 7.42) | 7.40 (7.36, 7.44) | 7.42 (7.37, 7.47) |
| Unknown | 2 | 2 | 0 | 0 |
| CPB lactates (mmol/L) | 1.88 (1.50, 2.50) | 1.88 (1.50, 2.78) | 1.90 (1.46, 2.28) | 1.79 (1.49, 2.65) |
| Unknown | 4 | 4 | 0 | 0 |
| CPB pCO2 | 34.3 (30.4, 37.1) | 34.7 (31.0, 36.9) | 34.3 (29.1, 37.9) | 33.2 (28.9, 35.2) |
| Unknown | 2 | 2 | 0 | 0 |
| CPB pO2 | 169 (144, 197) | 168 (145, 194) | 170 (130, 198) | 169 (150, 197) |
| Unknown | 2 | 2 | 0 | 0 |
| Post-surgery PaO2/FiO2 | 174 (90, 332) | 168 (85, 296) | 174 (106, 416) | 241 (102, 356) |
| Unknown | 3 | 2 | 0 | 1 |
| Post-surgery TA MPO (mU/ml) | 844 (322, 2,113) | 1,113 (440, 2,671) | 714 (266, 1,890) | 693 (240, 1,089) |
| Unknown | 28 | 19 | 4 | 5 |
| Post-surgery TA albumin (mg/ml) | 10 (7, 14) | 10 (6, 14) | 10 (8, 15) | 12 (10, 24) |
| Unknown | 27 | 18 | 4 | 5 |
| Post-surgery mechanical ventilation (h) | 36 (27, 73) | 34 (28, 76) | 30 (11, 54) | 54 (32, 74) |
| Post-surgery mechanical ventilation  > 48h | 65 (46%) | 40 (46%) | 16 (42%) | 9 (60%) |
| ICU stay (days) | 2.66 (1.65, 4.66) | 3.65 (1.65, 4.67) | 2.65 (1.65, 3.65) | 3.65 (2.66, 5.39) |
| Unknown | 1 | 0 | 1 | 0 |
| Length of stay (days) | 8.7 (6.6, 13.2) | 8.6 (6.6, 12.7) | 9.6 (6.6, 13.6) | 7.6 (6.7, 11.7) |
| Unknown | 1 | 0 | 1 | 0 |
| *^1^n (%); Median (IQR)* | | | | |

Legend: CPB: cardiopulmonary bypass; DHCA: deep hypothermic cardiac arrest; MPO: myeloperoxydase activity; ICU: intensive care unit; STAT : The Society of Thoracic Surgeons-European Association for Cardio-Thoracic Surgery score; TA: tracheal aspirates. Unknown: missing data. Continuous variables are expressed as median (interquartile range)
